# Water permeability of seed coats determines differences in dormancy between two orchid species

**DOI:** 10.1186/s12870-026-08982-0

**Published:** 2026-05-27

**Authors:** Anna Kaňáková, Edita Tylová, František Novák, Tomáš Figura, Stanislav Vosolsobě, Jan Ponert

**Affiliations:** 1 Department of Experimental Plant Biology, Faculty of Science, Charles University, Viničná 5, Prague, Czech Republic; 2 Institute of Botany, Czech Academy of Sciences, Průhonice, Czech Republic; 3 Prague Botanical Garden, Trojská 800/196, Prague, Czech Republic

**Keywords:** Dust seed, Germination, Permeability, Dormancy, Carapace, Scarification, Disinfection, In vitro

## Abstract

**Background:**

Seeds of mycoheterotrophic plants including orchids are extremely small and structurally reduced, with two layers of seed coats: large testa and a thin inner seed coat known as the carapace. Germination in many species is limited and often requires chemical pretreatment, suggesting that seed coat permeability could play an important role. However, the relative contributions of the testa and carapace to permeability and germination remain unclear, and it is unknown whether these structures may contribute to a physical dormancy. This study aimed to determine the structural basis of seed coat permeability and its role in regulating germination in two terrestrial orchid species with contrasting germination ability, *Dactylorhiza majalis* and *Epipactis helleborine*.

**Results:**

Histochemical and ultrastructural analyses showed that the testa is composed of lignified cell walls and lacks detectable cutin or suberin. In contrast, the carapace consists of several structurally distinct layers, some of them likely containing lipidic material. The overall organization of the carapace was similar in both species, but it was significantly thicker in *E. helleborine*, particularly in the inner autofluorescent layer, which remained largely resistant to chemical pretreatment. Permeability assays developed in this study demonstrated that the carapace represents the main barrier to water entry, and the proportion of permeable seeds closely corresponded to germination percentages in both species. In contrast, differences in testa permeability were relatively minor and could not explain the large interspecific differences in germination.

**Conclusions:**

Both seed coat layers, the outer testa and the inner carapace, act as barriers to water permeability in orchid seeds. However, the carapace constitutes the dominant barrier controlling water access to the embryo. The permeability of the carapace strongly influences germination ability and explains interspecific differences in germination response. Our findings provide direct evidence that a physical or combinational dormancy may occur even in structurally reduced dust seeds and significantly advance understanding of dormancy mechanisms in mycoheterotrophic plants.

**Supplementary Information:**

The online version contains supplementary material available at 10.1186/s12870-026-08982-0.

## Introduction

Timing of seed germination is one of the key processes in plant life and it is essential for the subsequent survival of seedlings. To time the onset of germination at a suitable place and time, plant seeds can combine different mechanisms: afterripening, dormancy and regulation of germination itself. These mechanisms can differ significantly between species and several types of dormancy have been identified in different species. However, not all species have been studied and especially the mechanisms of dormancy and regulation of seed germination in mycoheterotrophic plants are largely unknown. The majority of mycoheterotrophic plants have tiny dust seeds, usually with an undeveloped embryo and sometimes even without endosperm [1, 2]. Seed coats are composed of dead cells and while the outer seed coat (testa) is usually well developed with secondary cell walls [3–5], the inner seed coat (tegmen) usually develops by collapse of inner integument during seed development into a very thin layer without evident cellular structure which tightly surrounds the living parts of the mature seed [5, 6]. However, in some species, this layer is indistinguishable or even absent in mature seeds [7, 8]. The specific structure of mycoheterotrophic plant seeds begs the question of whether the mechanisms regulating dormancy and germination of these seeds can be similar to those operating in larger seeds of other plants.

The largest and most studied group of mycoheterotrophic plants are orchids (family Orchidaceae). Their seeds lack endosperm and the tegmen is traditionally called the carapace in this family. The testa of orchid seeds is composed mainly of cellulose [9, 10] and is generally lignified [11–13]. While some species contain only G/S-lignin, others can also contain C-lignin [11, 14] and the presence of unidentified polyphenols has been observed in several other species [5, 15, 16]. There are several reports about the presence of cutin in orchid testa. However, these are based on staining with Nile Red [5, 8, 15–17] which can also non-specifically stain lignin in plant tissues [18] and ATR-FTIR analysis did not support presence of cutin in testa [10], indicating that cutin is likely not present in orchid testa [8]. There are also reports about the presence of suberin in orchid testa [19, 20]. However, we have not found any experimental data supporting this statement, such as histochemical or analytical detection of suberin in orchid seeds. The carapace of orchid seeds can be lignified [6, 21] and can contain a layer strongly positively stained by Nile Red, thus likely cutinized [6, 15, 22, 23]. It has also been observed that the carapace probably contains polyphenols [15]. In some species, another layer of material strongly positively stained by Nile Red, thus possibly a cuticle, is present on the embryo surface [5, 8, 21].

There is substantial evidence that partial degradation of the testa, which is generally believed to be hardly permeable, can improve germination of various orchid species. These plants are usually propagated in vitro and disinfection of seeds with disinfection solutions prior to sowing frequently improves germination percentage, and some species even do not germinate without such a treatment [10, 17, 20, 24, 25]. There are two possible mechanisms by which seed disinfection could improve germination. The disinfection solutions could do chemical scarification of the seeds as these are corrosive solutions which likely penetrate seed coats [2, 17, 26, 27]. A second possible explanation is the removal of germination inhibitors. It has been observed that disinfection of orchid seeds can lead to degradation of endogenous abscisic acid [16]. It should also be noted that abscisic acid can be localized extracellularly outside the embryo [28]. It could therefore be hypothesized that the reduction in total ABA concentration in the seed after disinfection might be connected to degradation of the seed coats, which could release the inhibitor from the seed. Improvement of germination has also been achieved by mechanical scarification using vacuum [29], ultrasound [30, 31], removal of the testa [32], and by degradation of seed coats with enzymatic solutions: cellulase and maceroenzyme [29] or laccase [27]. It is unknown which mechanisms are responsible for permeabilization of seed coats under natural conditions; nevertheless, the role of symbiotic fungi is expected [17].

However, the requirement for permeabilization of seed coats described above is not present in all orchids and there are strong differences between species. While some germinate readily even without any degradation of seed coats, others do not germinate without intensive permeabilization and some do not germinate in artificial conditions at all (e.g. [17]). It is largely unknown what drives these differences and a combination of several factors is likely responsible. For example, some species require cold stratification [33, 34], some are highly sensitive to nitrates [35], and some are inhibited by light [17]. However, it has also been proposed that the thickness of the carapace might be responsible for differences in germination percentage between some European orchid species [36]. Nevertheless, this assumption has not been experimentally tested. Here we focused on two European terrestrial species with contrasting germination response, *Dactylorhiza majalis* which germinates relatively readily, even after very weak pretreatment, and *Epipactis helleborine* which germinates very poorly and requires intensive disinfection [33]. We aimed to test whether permeability of seed coats could be responsible for this difference, and to identify possible mechanisms of disinfection treatment on the permeability of seed coats. It should also be noted, that despite the numerous results indicating an importance of seed coats impermeability in preventing germination of orchid seeds, it is still questionable whether it could cause true physical or combinational dormancy caused by seed coat impermeability. A recent review on seed dormancy surprisingly classifies orchids as having “Morphophysiological” dormancy, noting that physical dormancy is unlikely to occur and requires further study [37]. We therefore also aimed to determine whether the mechanism could be classified as physical or combinational dormancy.

## Material and methods

### Plant material

**Fig. 1:**
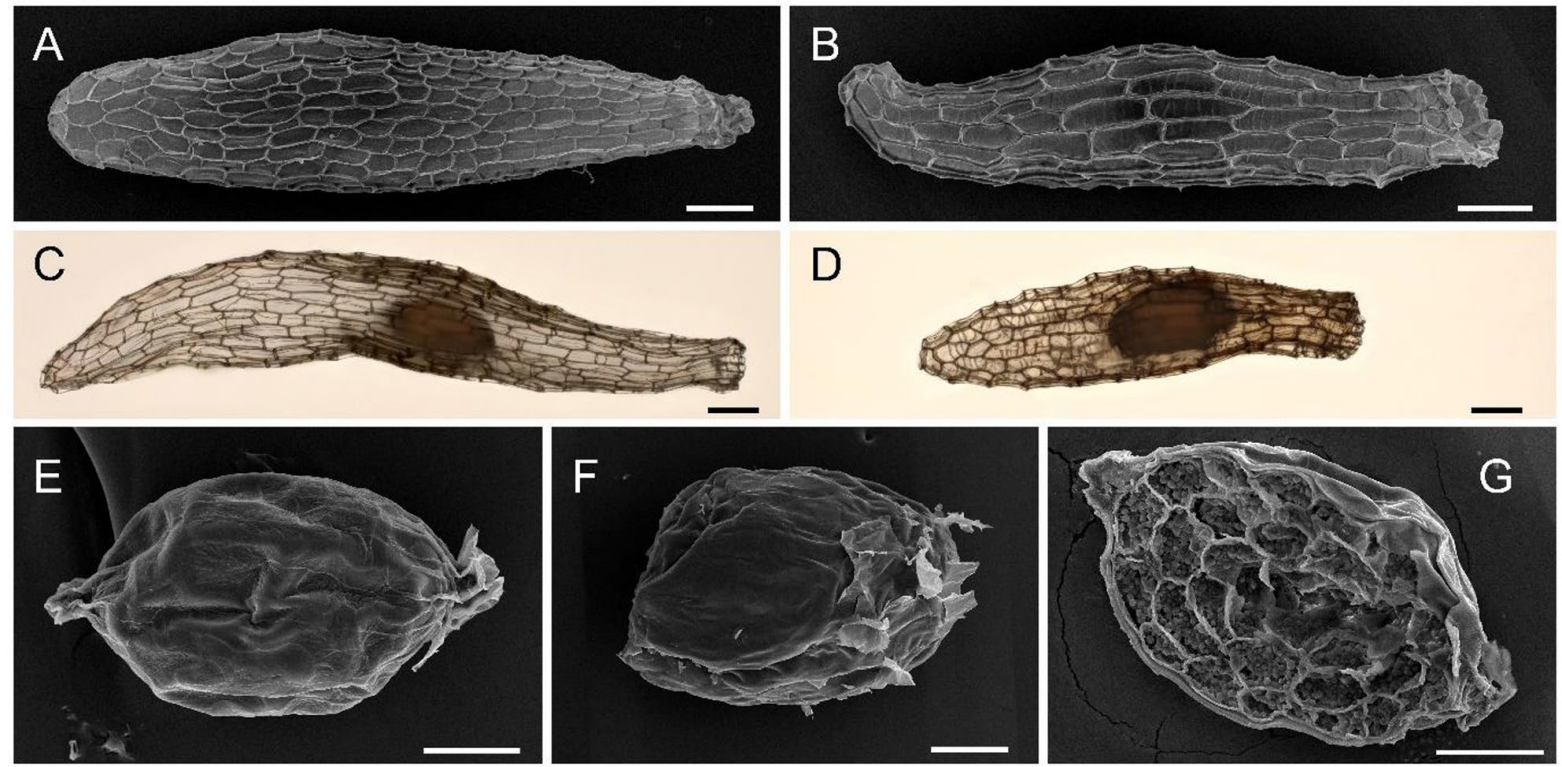
Seeds of the studied species. SEM images of the intact mature seeds of (**A**) *Epipactis helleborine* and (**B**) *Dactylorhiza majalis* showing surface structure of testa. Brightfield images of the seeds of (**C**) *E. helleborine* and (**D**) *D. majalis* show relatively small embryo inside the large testa. Close up SEM image of embryo covered in carapace (inner seed coat) of (**E**) *E. helleborine* and (**F**) *D. majalis*, removed from the testa. **G** SEM image of longitudinal section of *E. helleborine* embryo enveloped in the carapace. All pictures were taken without fixation or staining to avoid possible artifacts. Scale bars **A**-**D**: 100 μm, **E**-**G**: 50 μm

Two European terrestrial species with contrasting germination responses were compared: *Dactylorhiza majalis* and *Epipactis helleborine* (Table S1, images of the seeds in Fig. 1). The seeds were collected from ripe seed pods which had already started to open to ensure that the seeds were completely mature (Fig. 1). The seeds were placed in paper bags and stored in dry and dark place at room temperature no longer than one year.

### In vitro culture

The medium BM-2 (Himedia [38]), was supplemented with 0.5 g·L^− 1^ of activated charcoal. Medium pH was adjusted to 5.8 with NaOH, autoclaved at 144 kPa, 121 °C (Tuttnauer 2540 EK-N) for 20 min and poured into 9 cm plastic Petri dishes. The seeds were disinfected in 5 ml syringes and sown as a suspension of disinfected seeds in sterile deionised water as described previously [39, 40] which resulted in 50 to 300 seeds in each Petri dish. Ca(OCl)_2_ solution was prepared by dissolving 20 g of chlorinated lime (Kittfort, Czech Republic) in 100 ml deionised water, filtered through filter paper and a drop of Tween 20 was added following Figura et al. [2]. This solution was used within 60 min after filtering. Petri dishes were sealed with air permeable foil (Parafilm M). In the case of *D. majalis*, the cultures were incubated in the dark at 20 °C. In the case of *E. helleborine*, which requires cold stratification after initial incubation at regular temperature [17, our data], cultures were incubated one month at 20 °C, three months at 4 °C and then at 20 °C. Cultures were observed using a Zeiss Stemi 305 stereomicroscope and germination percentage was counted manually monthly, except of stratification period. The seeds without embryo or with obviously undeveloped embryo were excluded from counting as these were unable to germinate. Only well-developed seeds with ruptured testa were considered as germinated seeds following Figura et al. [2] and all counts represent total number of germinated seeds at the respective time (are cumulative). Pictures of seedlings were taken with a Canon EOS 60D equipped with a Canon EF 100 mm f/2.8 L macro lens.

Seeds of both species were pretreated with 70% ethanol, as this is widely used common practice which is believed to improve disinfection (e.g. [17, 35]), and treated with Ca(OCl)_2_ solution for different times to determine differences in their germination response. In addition to testing different times of exposition to the Ca(OCl)_2_ solution, we also included one experimental variant without ethanol pretreatment to verify whether the ethanol pretreatment affects seed germination With the seeds of *E. helleborine*, which germinate very poorly, we also tested another two approaches which are sometimes used for difficult-to-germinate orchid species: (i) treatment with NaOCl solution instead of Ca(OCl)_2_ solution (e.g. [17, 41]), and (ii) pretreatment with 2% H_2_SO_4_ before treatment with Ca(OCl)_2_ solution (e.g. [40, 42, 43]). Each experimental variant was prepared in seven or eight replications (Petri dishes).

### Anatomical analyses

Anatomical analyses of seed coats were performed on intact seeds, cryosections, and sections of resin-embedded seeds. To make the crysections, seeds were transferred into Cryo-Gel (Leica microsystems, Germany, Wetzlar) and frozen at -63 °C. Sectioning into 20 μm thick sections was performed at -20 °C using Shandon cryotome, and subsequent staining was performed directly on microscope slides coated with alum gelatin adhesive [44]. Resin embedding involved seed fixation in 4% paraformaldehyde in 0.1 M phosphate buffer (pH = 7.1), dehydration in ethanol series, and gradual embedding into LR White resin. Resin-embedded samples were sectioned (3 μm) on Leica RM 2265 microtome. Staining of intact seed was carried out in a syringe into which the staining solutions were gradually sucked in. To detect lignin in testa, intact seeds or sections were stained with 1% (w/v) phloroglucinol in 18% aq. HCl according to Soukup [45] or with 0.1% solution (w/v) of berberine hemisulfate counterstained with 0.05% Crystal Violet for 10 min according to Brundrett et al. [46] and Soukup [45]. Sections were mounted into 65% glycerol, and observed in Olympus BX51 microscope (Olympus, Japan, Tokyo) with a Nikon Digital Sight DS-U3camera using UV-induced fluorescence (filter cube Olympus U-MWU) for berberine-stained sections. Lipidic substances were detected with 0.01% (w/v) Sudan Red 7B or 0.01% (w/v) Fluorol Yellow 088 according to Brundrett [18] and Soukup [45], with 0.01% (w/v) Auramine O in 0.05 M Tris buffer (pH 7.2) in combination with 0.1% (w/v) Calcofluor white M2R staining according to Buda [47], and with Nile Red using a modified procedure of Yeung et al. [5]. Briefly, the stock solution of Nile Red in acetone (1 mg ml^− 1^) was diluted 1:100 in 50% glycerol prior to staining and sections were stained in the dark for 10 min. All stained sections were mounted in 50% glycerol. Sections stained with Fluorol Yellow 088 were observed in Olympus BX51 microscope (filter block Olympus U-MWU) or confocal microscope Leica SP8 (excitation 405 nm, emission 529–573 nm). Auramine O was excited with 458 nm and emission was collected between 491 and 563 nm, Calcofluor White M2R was excited with 405 nm and emission collected between 415 and 448 nm. Sections stained with Nile Red were observed in Olympus BX51 microscope (filter block Olympus U-MWG).

Permeability of testa (outer seed coat) was tested with a 0.1% solution (w/v) of PTS (8-hydroxy-1,3,6-pyrenetrisulfonic acid trisodium salt) which can be directly visualised with fluorescence [48, 49]. The aqueous solution of this apoplastic tracer was applied to seeds and its presence below testa was directly observed with fluorescence excited with blue light using confocal microscope Leica SP8 (excitation 405 nm, emission 513–540 nm) and Olympus BX51 microscope (filter block Olympus U-MWB). 20–50 seeds were observed from each variant. Seeds in which the solution entered under the testa immediately were categorized as permeable. Seeds in which the solution entered under the seed coat within 30 min after staining were categorized as partially permeable. Seeds in which the solution did not enter under the outer seed coat even after this time were categorized as impermeable.

Permeability of the carapace (inner seed coat) was impossible to observe with the PTS solution as the embryos are optically very dense. It was therefore tested with a 0.1% solution of berberine hemisulfate. This stain is soluble in water and stains tissues, which came into contact with its aqueous solution [45, 46]. To stimulate the penetration of staining solution through the testa, we treated seeds in a staining solution for one hour after degassing with a vacuum for one hour. The intact seeds were then mounted into 65% glycerol and observed with a confocal microscope LeicaSP8 (excitation 405 nm, emission 530–570 nm) at the median optical section of embryo (20 seeds from each variant). Seeds with stained embryos (in the median optical section) were categorised as permeable. Seeds with less than a half of embryo stained were categorized as partially permeable and seeds without detectable staining as impermeable.

Ultrastructural details were observed using transmission electron microscopy (TEM). The seeds were fixed in 2,5% glutaraldehyde in the cacodylate buffer (0.1 M, pH 7.2), post-fixed in 2% osmium tetroxide in the same buffer, dehydrated in ethanol series, and embedded in LR White resin. Ultrathin sections were contrasted with uranyl acetate and lead citrate according to Reynolds [50]. The sections were observed in the microscope JEOL JEM-1011 with camera Veleta CCD.

Structural details were observed using scanning electron microscopy (SEM). The dry mature seeds were hand-sectioned without fixation, mounted on stubs with a carbon conductive tape using micromanipulatory needles, dried over silica-gel overnight, coated with approximately 5 nm Au in a Bal-Tec SCD 050 sputter-coater and observed with JEOL 6380 LV microscope at an accelerating voltage of 15 kV. To characterise the width of carapace, we used median longitudinal sections of the embryos and measured the thickness of the whole layer in the median part of the embryo. We performed three measurements from each seed and measured ten seeds from each species.

### Statistical analyses

The germination percentage of axenic in vitro cultures was analysed using a linear model to assess the effects of species, seed pretreatment, and the time of counting after sowing. The final model included species, pretreatment, and counting time as fixed factors, as well as the interaction between species and pretreatment. This interaction was included because preliminary analyses indicated that the effect of pretreatment on germination differed between species. Other potential interactions were not included in the final model, as they did not improve model fit according to AIC or did not explain a substantial portion of the variance. To meet the assumptions of normality and homogeneity of variance, germination percentages were arcsine square root transformed prior to analysis. Effect sizes were calculated using partial eta-squared (η²) to quantify the relative contribution of each factor. All analyses were performed in R version 4.5.2 [51] using the lm function, and effect sizes were computed with the effectsize package [52].

The width of carapace was analysed using a nested ANOVA to evaluate differences between species while accounting for the hierarchical structure of the data. The species was specified as a fixed factor, and the seed identity was specified as a random factor nested within species, as multiple measurements were obtained from each seed. The analysis was performed in R version 4.5.2 [51] using the *aov()* function with the model formula *width ~ spec/seed*. In this model, the term *spec: seed* represents variation among seeds within species and was used as the denominator mean square for testing the effect of species. F-statistics were calculated from the corresponding mean squares, and p-values were obtained from the F-distribution using the appropriate degrees of freedom for the nested design. The assumptions of normality and homogeneity of variance were assessed using diagnostic plots of model residuals.

## Results

### Germination in vitro

**Fig. 2:**
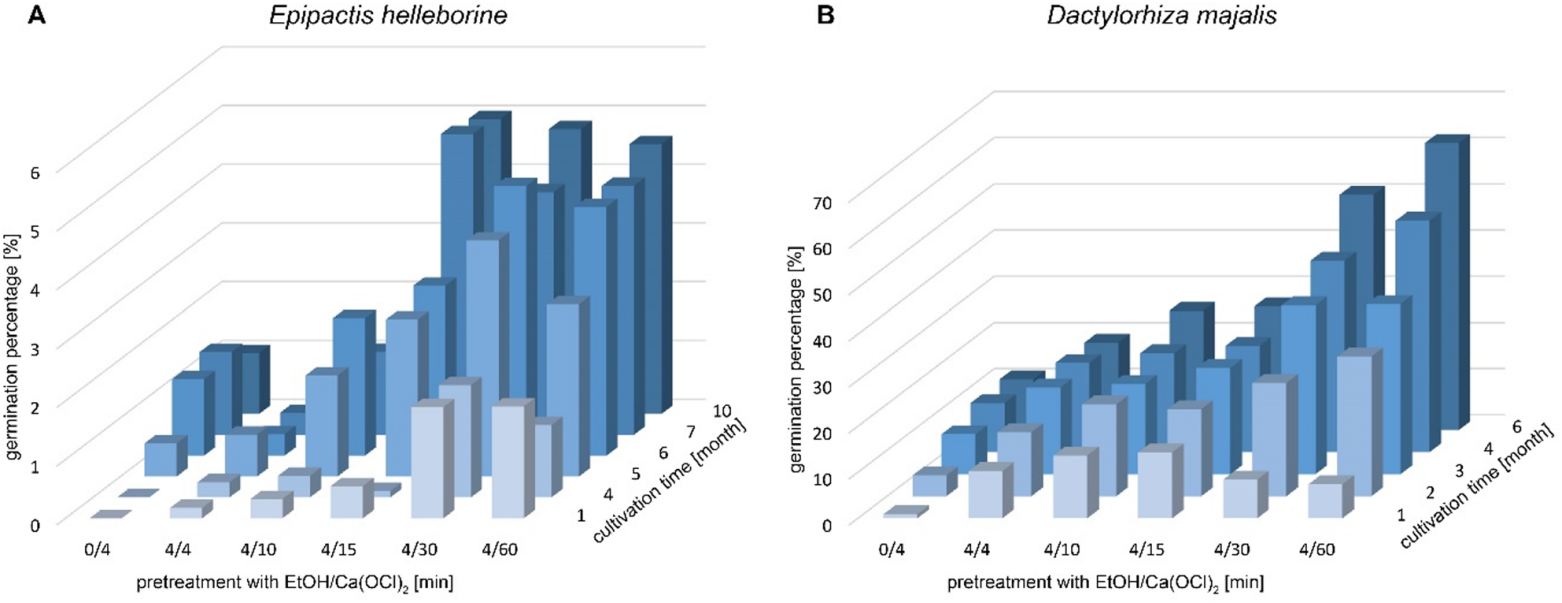
Effect of different treatments with routinely used CaOCl_2_ solution on axenic in vitro seed germination of two model species: (**A**) *Epipactis helleborine*, (**B**) *Dactylorhiza majalis*. Numbers on the x axis describe time of pretreatment with ethanol (EtOH) / time of pretreatment with CaOCl_2_ solution. The germination percentage differed significantly between species (*p* < 0.001), seed pretreatments (*p* < 0.001) and time of counting after sowing (*p* < 0.001), according to a linear model applied to arcsine-transformed germination percentages. *E. helleborine* was stratificated by low temperature between 1^st^ and 4^th^ month

We used two European terrestrial species with contrasting germination responses (images of the seeds in Fig. 1) and our results confirmed that *Dactylorhiza majalis* germinates readily, even after very weak pretreatment, while *Epipactis helleborine* germinates very poorly. Pretreatment of seeds with ethanol improved germination in both species (Fig. 2) and seeds of both species germinated best after longest treatment with Ca(OCl)_2_ solution (effect of seed treatment: *F*_5,452_ = 83.1, *p* < 0.001, *η*^2^ = 0.48). The proportion of germinated seeds also increased with time of incubation after sowing in both species (effect of counting time: *F*_5,452_ = 61.8, *p* < 0.001, *η*^2^ = 0.41). However, there were significant differences between species in achieved germination percentages and time to germination (effect of species: *F*_1,452_ = 2386, *p* < 0.001, *η*^2^ = 0.84). *D. majalis* achieved high germination percentages up to 60%, while *E. helleborine* only about 5%. *D. majalis* was able to germinate in all experimental variants at least about 10% (Fig. 2), while in *E. helleborine* only individual seeds germinated after Ca(OCl)_2_ treatment shorter than 15 min (Fig. 2). Our model also identified significant interaction between species and treatment (*F*_5,452_ = 22.0, *p* < 0.001, *η*^2^ = 0.20), indicating that the effect of treatment differed between species. The model explained 88% of the variation in germination (*R*^2^ = 0.877). Additional experiments revealed that the treatment of *E. helleborine* seeds with NaOCl solution instead of Ca(OCl)_2_ (Fig. S1) or the pretreatment with H_2_SO_4_ (Fig. S2) did not improve germination.

### Structure of testa

**Fig. 3:**
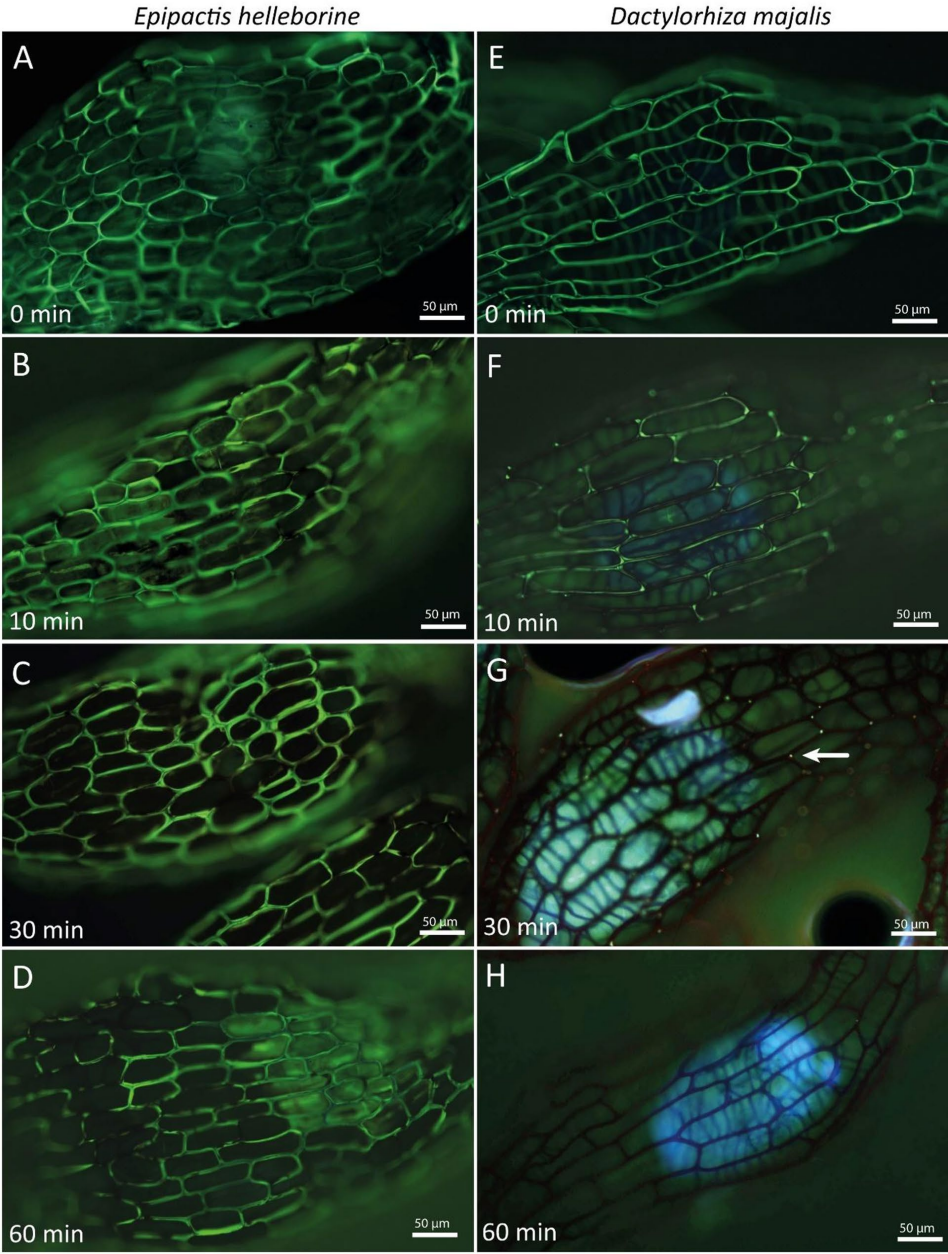
Detection of lignin in testa of intact seeds after pretreatment with ethanol (4 minutes) and Ca(OCl)_2_ treatment of different duration (indicated in the pictures) in (**A**-**D**) *Epipactis helleborine* and (**E**-**H**) *Dactylorhiza majalis*. **A**-**H** berberine hemisulfate and Crystal Violet staining, UV-excited fluorescence of lignified cell walls visible in yellow. Arrow marks an area of the cell wall where lignin remains detectable (**G**). Whole-mount preparations of seeds

We detected lignin in testa of untreated mature seeds of both species (Fig. 3). In *E. helleborine*, lignin was resistant to Ca(OCl)_2_ treatment and remained well-detectable with berberine staining even after 60 min of Ca(OCl)_2_ treatment (Fig. 3A-D). In *D. majalis*, even relatively short treatment with Ca(OCl)_2_ solution resulted in patchy degradation of lignin and after 60 min of Ca(OCl)_2_ solution, no lignin was detectable (Fig. 3E-H).

**Fig. 4:**
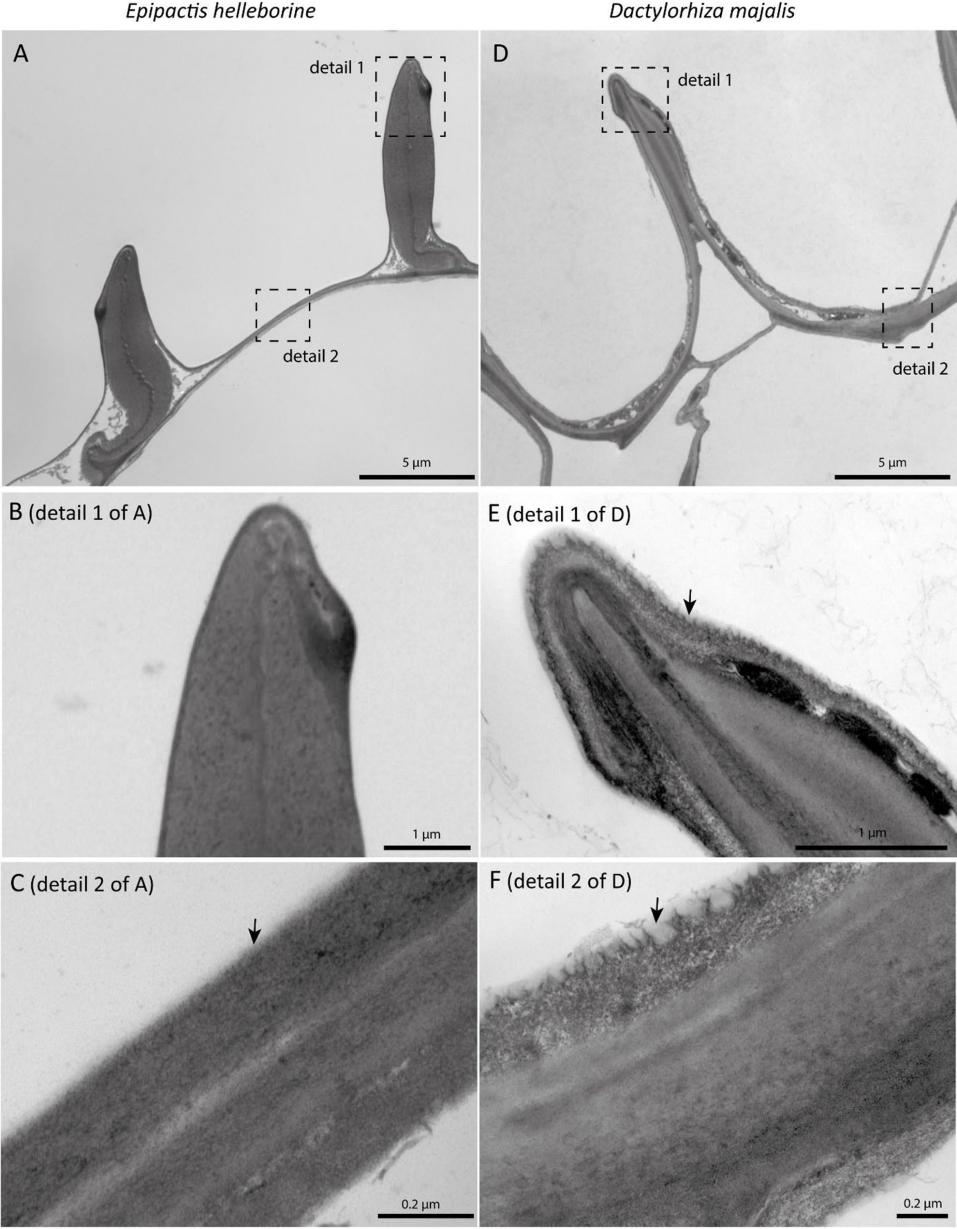
Ultrastructure of testa of the dry seeds untreated with Ca(OCl)_2_ in (**A**-**C**) *Epipactis helleborine* and (**D**-**F**) *Dactylorhiza majalis*. **C**, **F** Outer surface is oriented in images in upper position. **E**, **F** Arrow indicates a distinct layer at the surface of testa. Transmission electron microscopy

Histochemical staining with Sudan Red 7B and with Fluorol Yellow 088 both did not indicate the presence of lipidic substances in testa of both tested species. However, ultrastructural analysis showed that *D. majalis* has a distinctive layer of unknown composition on the outer surface of the testa outer periclinal cell walls (Fig. 4D-F). A similar layer was observed in *E. helleborine* but was less distinct (Fig. 4A-C).

**Fig. 5:**
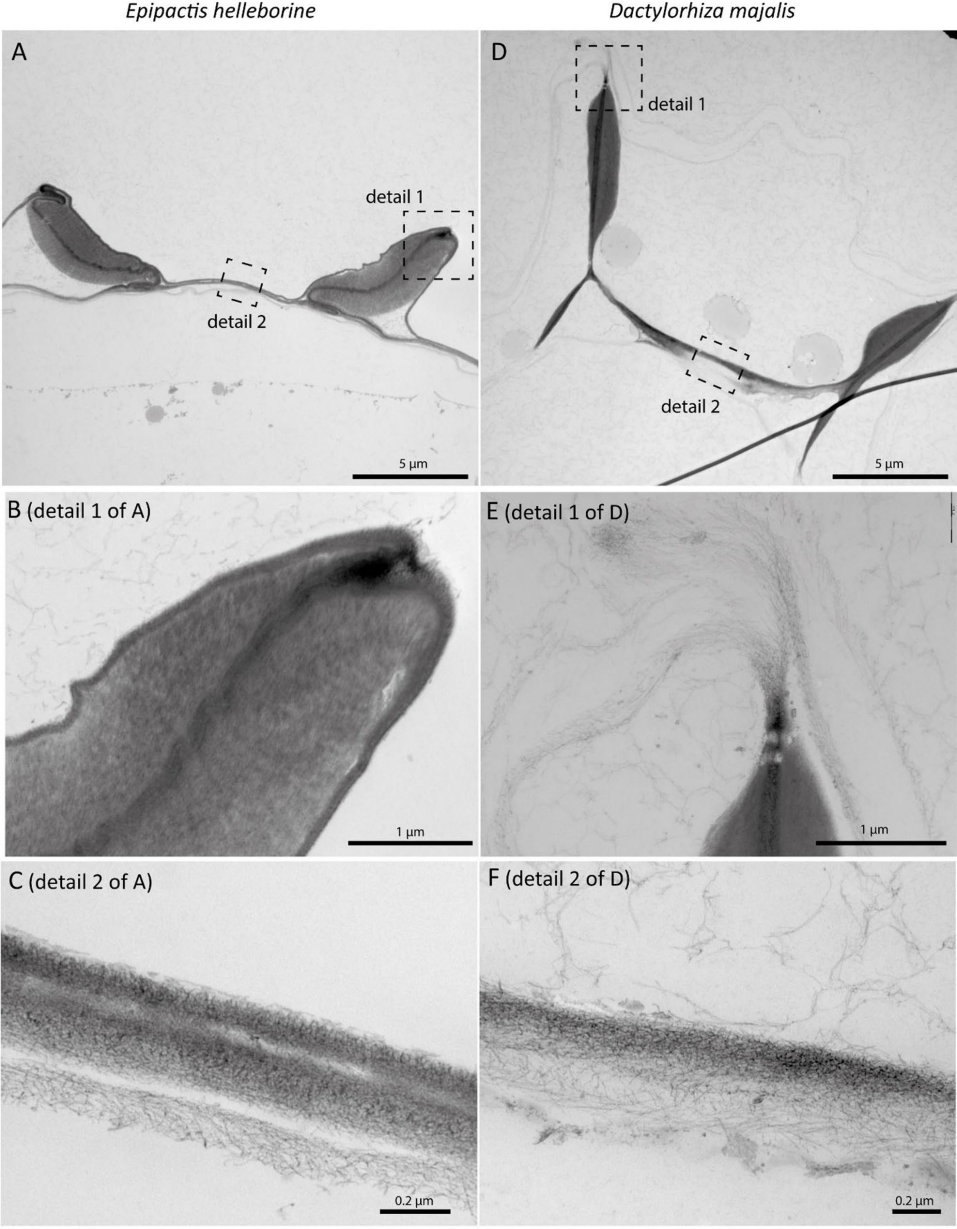
Ultrastructure of testa of the seeds treated with EtOH (4 min) and CaOCl_2_ (60 min) in (**A**-**C**) *Epipactis helleborine* and (**D**-**F**) *Dactylorhiza majalis*. **C**, **F** Outer surface is oriented in images in upper position. Note loosening in periclinal cell walls. Transmission electron microscopy

In both species, the outer cell layer of testa has thick anticlinal cell walls and cells are compressed. The outer periclinal walls are therefore in close proximity to the inner tangential walls (Fig. 4A, B). The Ca(OCl)_2_ treatment resulted in a visible loosening of the testa cell walls of both species, but this was more pronounced in *D. majalis* (Figs. 4 and 5).

### Structure of carapace and the surface of embryo

**Fig. 6:**
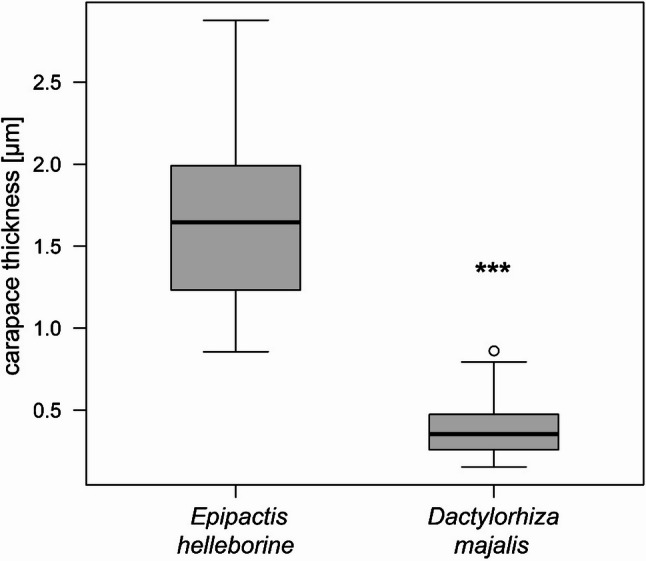
The thickness of carapace. Seeds untreated with fixation were manually cut and observed in SEM. Ten seeds from each species were measured and three measurements were done from each seed. Nested ANOVA (*p* < 0.001)

**Fig. 7:**
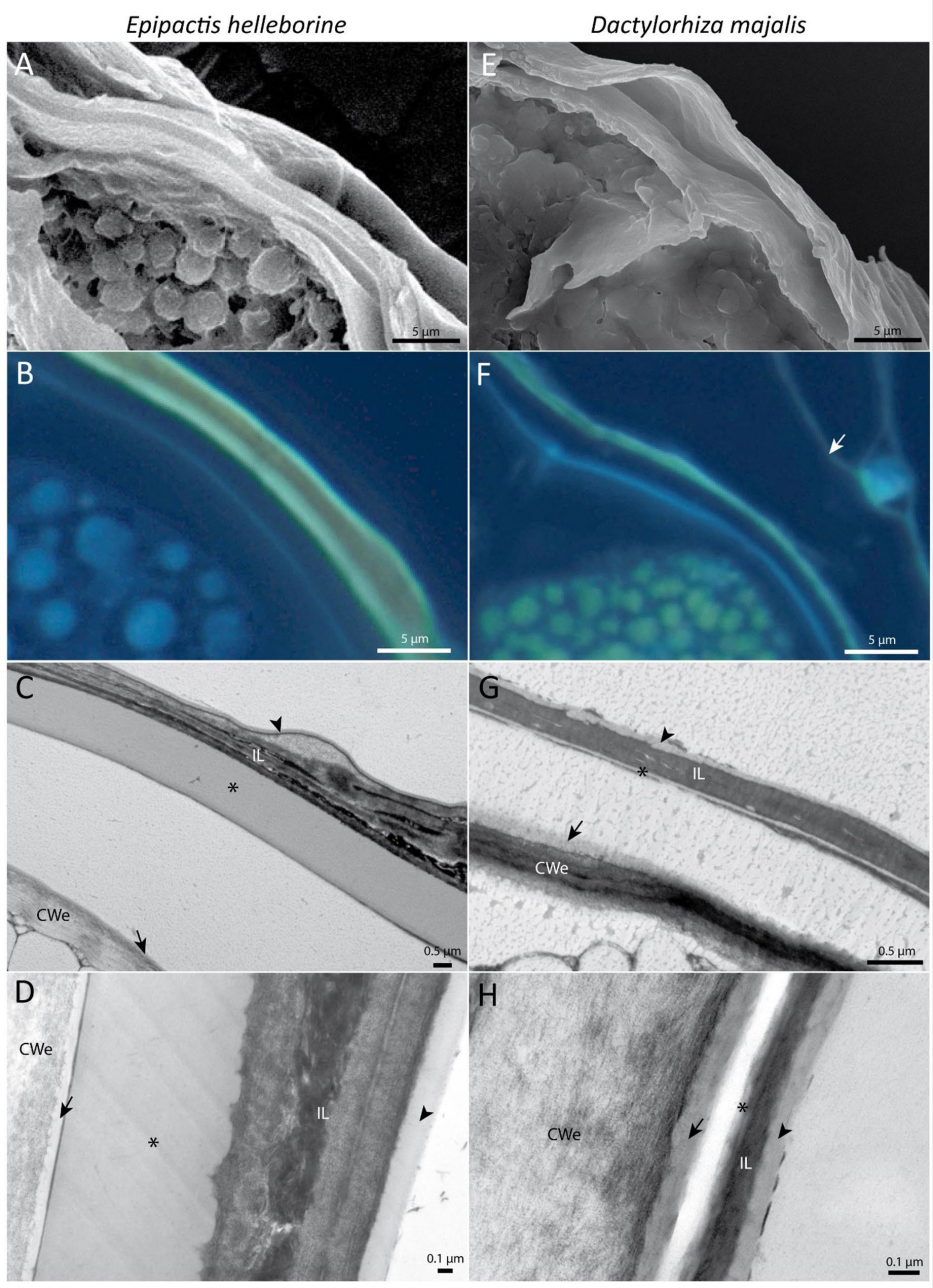
Structure of the carapace (inner seed coat) and the surface of the embryo proper of native seeds. **A**-**D** *Epipactis helleborine* and (E-H) *Dactylorhiza majalis*. **A**, **E** Ultrastructure of the carapace (scanning electron microscopy). **B**, **F** UV-excited autofluorescence of carapace on sections of resin-embedded seeds. White arrow indicates the testa (outer seed coat). **C**, **D**, **G**, **H** Ultrastructure of the carapace (transmission electron microscopy). Arrow indicates a distinct layer at the outer surface of the outer cell wall (CWe) of the embryo in both species, IL – inner layer of the carapace, an arrowhead indicates a distinct layer at the surface of carapace, an asterisk indicates a thick homogenous layer in the carapace of *E. helleborine* and similar thinner layer in carapace of *D. majalis*

**Fig. 8:**
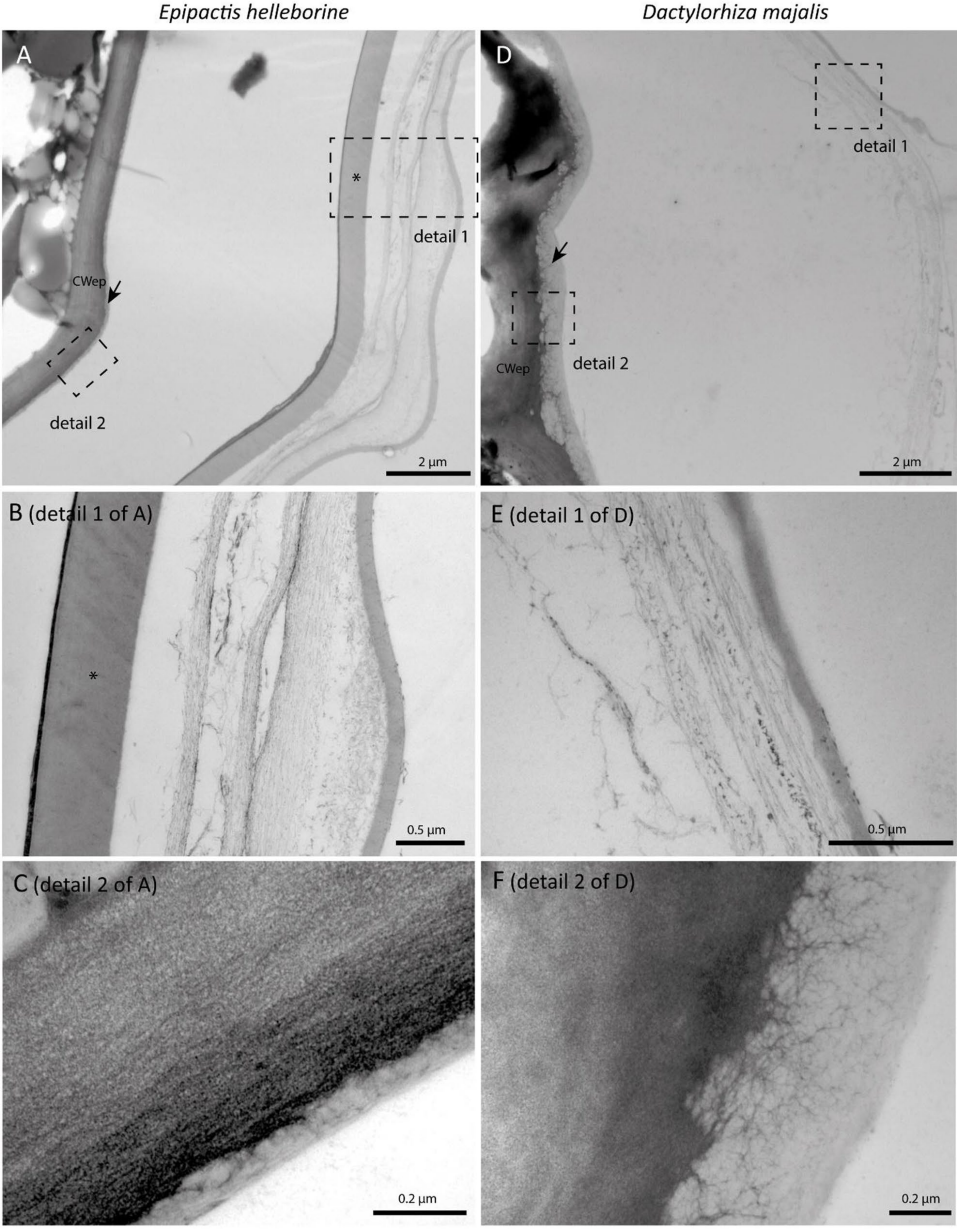
Ultrastructure of carapace (inner integument) of seeds treated with ethanol (4 min) and Ca(OCl)_2_ solution for 60 min to improve permeability. **A**-**C** *Epipactis helleborine* and (**D**-**F**) *Dactylorhiza majalis*. **A**, **D** Arrow indicates a distinct layer at the outer surface of the outer cell wall (CWe) of the embryo. **A**-**B** Asterisk indicates a thick homogenous layer in the carapace of *E. helleborine*. **C**, **F** Detail 2 shows the surface layer of the embryo, which remains compact. Transmission electron microscopy

**Fig. 9:**
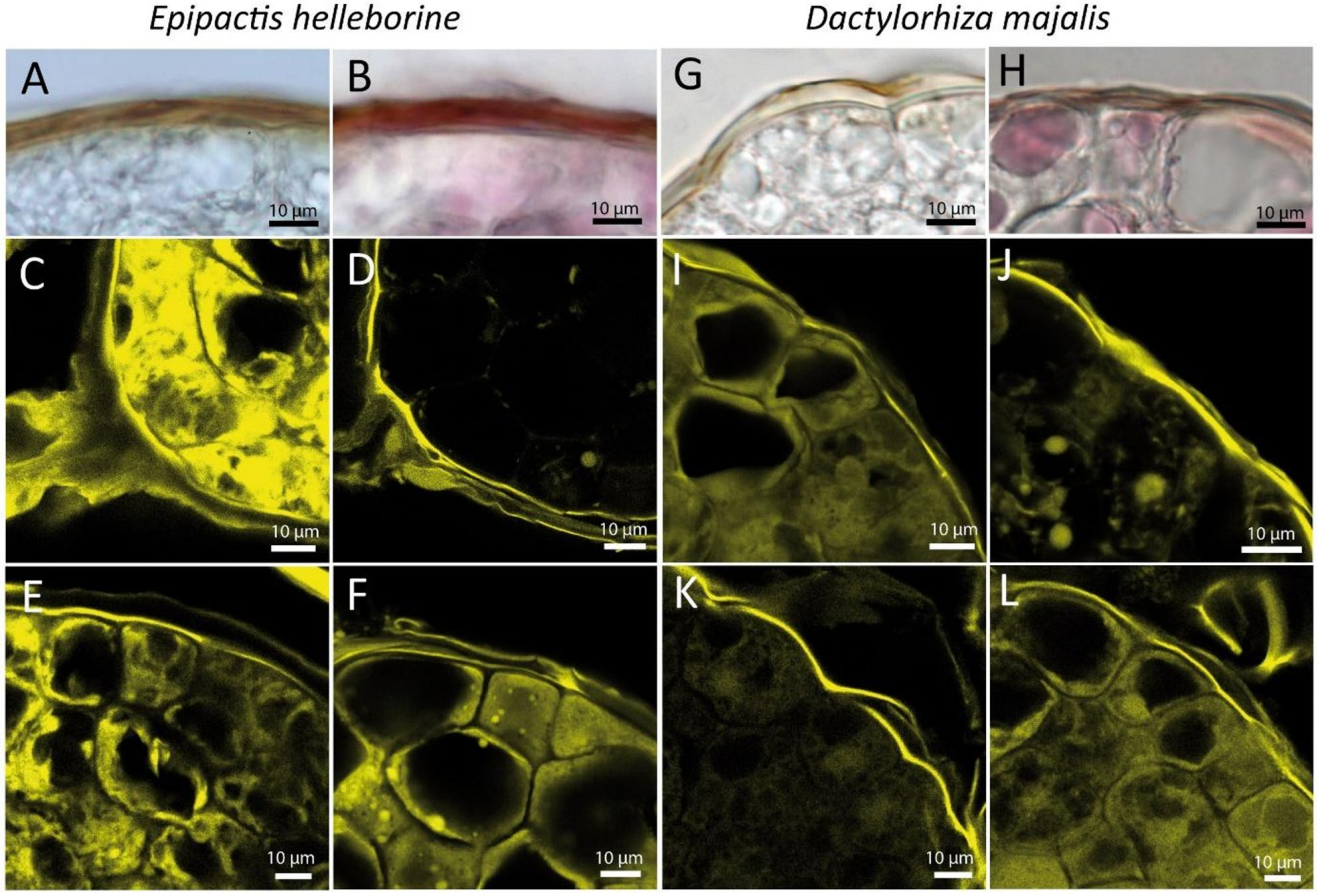
Histochemical detection of lipidic material in carapace in (**A**-**F**) *Epipactis helleborine* and (**G**-**L**) *Dactylorhiza majalis*. **A**, **B**, **G**, **H** Sudan Red7B staining in dry untreated seeds (brightfield). **A**, **G** negative controls and (**B**, **H**) sections stained with Sudan Red7B,. Fluorol Yellow staining in dry untreated seeds (**D**, **J**) and in seeds treated with EtOH (4 min) and Ca(OCl)_2_ solution for 60 min (**F**, **L**). Negative controls for dry untreated seeds (**C**, **I**) and for seeds treated with EtOH (4 min) and Ca(OCl)_2_ solution for 60 min (**E**, **K**). **A**-**L** Cryosections (20 μm). **C**-**F**, **I**-**L** confocal images (excitation 405 nm, emission 529–572)

**Fig. 10:**
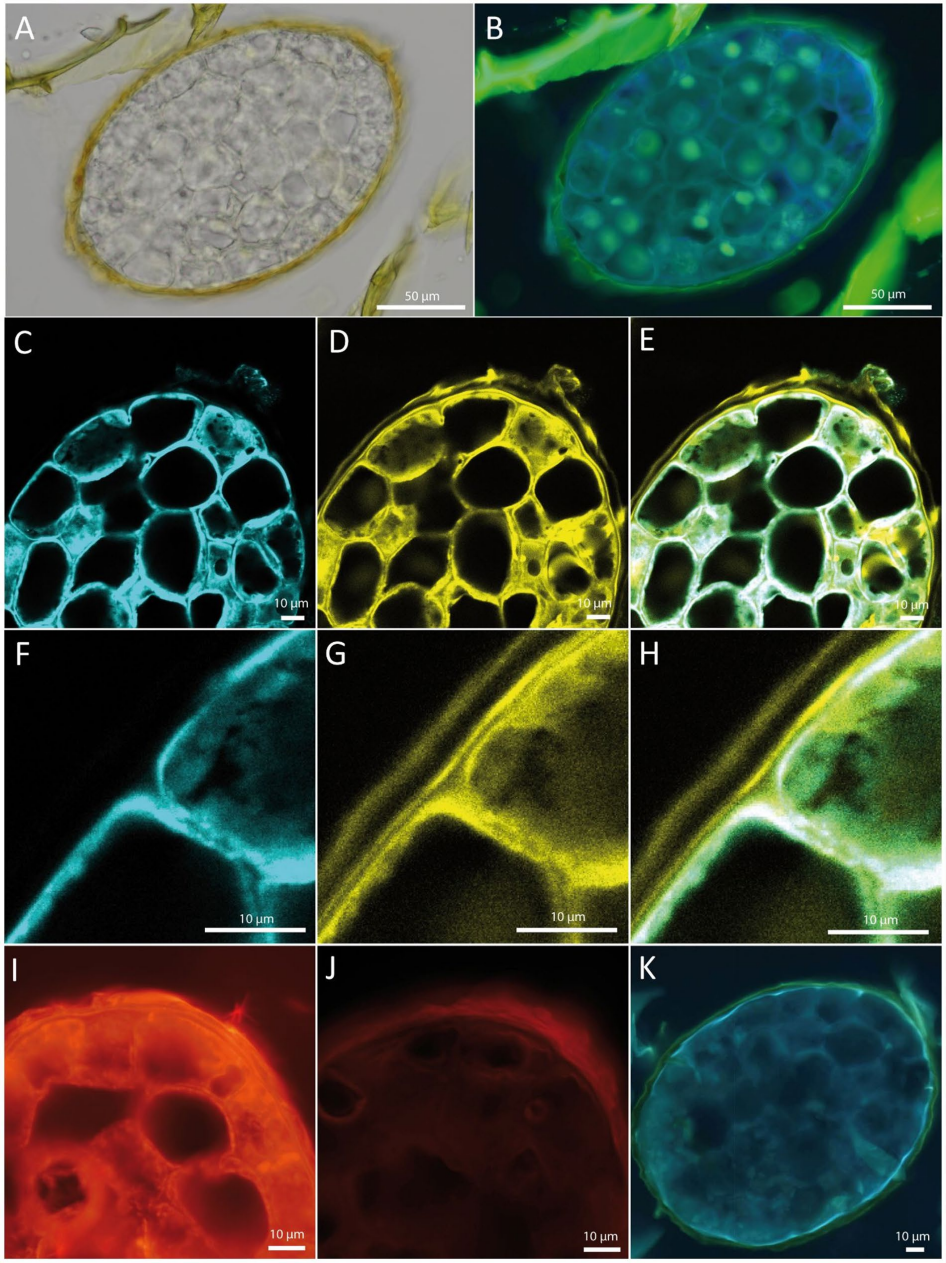
Histochemical staining of dry untreated seeds of *Epipactis helleborine*. **A**, **B** Auramine O/Calcofluor white M2R, (**A**) brightfield and (**B**) UV-excited fluorescence. **C**-**H** Auramine O/Calcofluor white M2R, confocal images. **C**, **F** Channel 1 showing Calcofluor white M2R fluorescence (emission 415–448 nm), (**D**, **G**) Channel 2 showing Auramine O fluorescence (emission 491–563 nm), (**E**, **H**) merge of channels 1 and 2. **I** Nile Red staining, green-light-excited fluorescence (**I**). **J**, **K** Negative controls (unstained sections), (**J**) Green-light-excited autofluorescence, (**K**) UV-excited fluorescence. **A**-**K** Cryosections (20 μm)

The SEM images of sectioned seeds unaffected with fixation (Fig. 1G) revealed that the carapace of *E. helleborine* is wider than that of *D. majalis* (Fig. 6) (nested ANOVA: *F*_1,18_ = 331.35, *p* < 0.001), while there is also significant variation among seeds within species (*F*_18,40_ = 5.77, *p* < 0.001). In both species, an autofluorescent layer is present at the outer surface of the globular embryo proper (Fig. 7B, F) and ultrastructural analysis revealed the presence of a distinct layer at the outer surface of the outer cell wall (Fig. 7C, D, G, H). This layer seems unaffected by Ca(OCl)_2_ treatment (Fig. 8) and resembles a cuticle layer in TEM images (Figs. 7 and 8). Histochemical tests (Auramine O, Fluorol Yellow, Sudan Red7B, or Nile Red) indicated the presence of lipidic material in this layer, but the signal is generally difficult to distinguish from background autofluorescence or tissue colour (Figs. 9 and 10).

Ultrastructural analysis of carapace (inner integument) revealed a complex structure of several layers (Fig. 7). The carapace and also the layers are wider in *E. helleborine* than in *D. majalis* and Ca(OCl)_2_ treatment resulted in loosening of internal layers which are likely composed of polysaccharides (Fig. 8). At the outer surface of carapace, there is an autofluorescent layer, structurally homogenous in TEM images, which seems to be mildly affected by Ca(OCl)_2_ treatment. Moreover, a thick structurally homogenous strongly autofluorescent layer is present in the inner surface of the carapace in *E. helleborine* (Fig. 7B-D), which remains optically intact after Ca(OCl)_2_ treatment (Figs. 7B-D and 8A). We detected this layer also in *D. majalis*, but not in all seeds and it is generally thinner (Fig. 7F-H).

### Permeability of testa

**Fig. 11:**
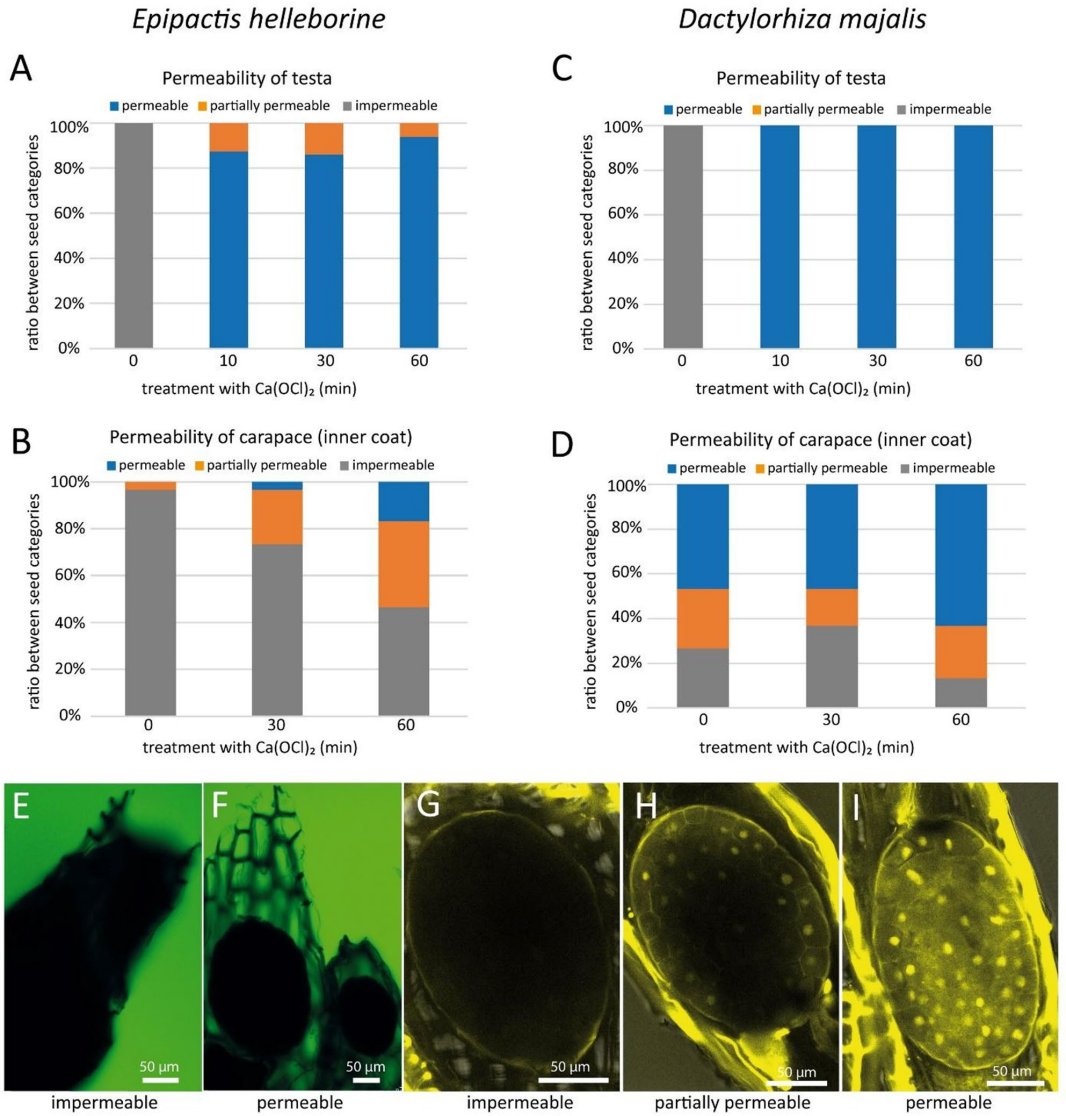
Effect of Ca(OCl)_2_ treatment on permeability of testa (outer seed coat) and carapace (inner coat) in (**A**-**B**) *Epipactis helleborine* and (**C**-**D**) *Dactylorhiza majalis*. **A**, **C** Differences in proportions of seeds with different permeability of testa according to permeability test with PTS. **B**, **D** Differences in proportions of seeds with different permeability of carapace according to permeability test with berberine hemisulfate. **E**, **F** Permeability test of testa with PTS in *D. majalis.* Representatives of two permeability categories of seeds. Optical sections from confocal microscope (excitation 405 nm, emission 513–540 nm). Green colour represents aqueous solution of PTS, which does not enter seed coats in untreated seeds. Whole-mount preparations of seeds. **G**-**I** Permeability test of carapace with berberine hemisulfate staining (60 min) in *D. majalis.* Representatives of three permeability categories of seeds, confocal images (excitation 405 nm, emission 530–570 nm). Whole-mount preparations of seeds

Dry untreated seeds were not permeable and it was obvious that aqueous solution of PTS apoplastic tracer is repelled from their hydrophobic surface in both species. Seeds treated with Ca(OCl)_2_ solution were permeable in both species and the staining solution filled the space between testa and carapace (Fig. 11A, C, E, F). In the seeds of *D. majalis*, the solution entered this space immediately after contact with seeds. In *E. helleborine*, some proportion of seeds did not allow the staining solution to penetrate immediately, but they became stained after 30 min incubation in PTS tracer (Fig. 11A, C).

### Permeability of carapace

The permeability of carapace was tested by application of aqueous solution of berberine hemisulphate and monitoring the staining inside the embryo by confocal microscopy. In both species, there was some proportion of seeds without detectable berberine staining of the embryo after 1 h of treatment with Ca(OCl)_2_ solution (Fig. 11G). We considered them impermeable and their frequency was much higher in *E. helleborine* than in *D. majalis* (Fig. 11B, D). Untreated seeds of *E. helleborine* were mostly impermeable while nearly 50% of those of *D. majalis* were partially or fully permeable with detectable berberine staining of embryo (Fig. 11B, D). Treatment with Ca(OCl)_2_ solution increased the proportion of permeable seeds (Fig. 11H, I) in both species, especially the long treatment for 60 min (Fig. 11B, D). It should be noted that some part of the observed difference of permeability could be caused by the testa, as we studied whole seeds. However, the testa allows water permeation relatively easily and even short treatment with hypochlorite solution leads to nearly complete permeability. The observed permeability of the carapace is much lower. Moreover, to eliminate the effect of testa, we pretreated the seeds subjected to the test of carapace permeability with a vacuum which helps water to enter below testa even without treatment with a hypochlorite solution. We therefore propose that the observed differences in carapace permeability are only slightly influenced with the presence of testa.

## Discussion

Our data suggest that the testa is composed of lignified cell walls. This has been observed also in other orchid species [11–13]. We did not detect cutin by our histochemical tests, which could support the view that reports about its presence in orchid testa are based on non-entirely specific staining with Nile Red [10, 18] and require further assessment. We also did not detect suberin, which had been reported from orchid testa without support in experimental data [9, 15, 19].

In the carapace, we detected several layers of different ultrastructure. Two of them are autofluorescent under UV excitation and they are visibly structurally distinct from other thicker, mostly polysaccharidic layers at TEM. Standard histochemical tests (Nile Red, Fluorol Yellow, or Auramine O) did not prove the presence of lipidic material in these layers with certainty, partially due to strong autofluorescence, but the presence of lipidic substances, likely cutin, has been reported in the carapace of several orchid species [5, 6, 15, 16, 21]. It is therefore likely that cuticular layers can be a general feature of the carapace, although difficult to detect due to their tiny dimensions. We also detected a layer of similar ultrastructure on the surface of the embryo proper. Such a layer has already been detected in *Cymbidium sinense*, *Cypripedium formosanum*, and *Paphiopedilum delenatii* and described as lipidic material or cuticle [5, 15, 21], so it could likely be widespread in orchids.

We can try to relate the difference between the ability of *D. majalis* and *E. helleborine* to germinate to the structural features of their seeds. The structure of testa did not differ substantially between these species, but its lignification seems to be significantly more resistant to the disinfection treatment in *E. helleborine*. This is reflected in slower permeation of water below the testa in this species, but the difference in testa permeability between the species is relatively weak. The testa of *E. helleborine* remains partially lignified after treatment with a disinfectant solution, but allows water to pass through. Thus, the interspecific differences in delignification can explain only a small part of the observed difference in germinability between the species and are likely not the main mechanism blocking germination of *E. helleborine*. We may therefore propose that the testa is not the only factor responsible for the considerable interspecific difference.

The structure of carapace is similar between the two studied species, composed of three distinct layers, but the carapace is significantly thicker in *E. helleborine*. Especially the inner autofluorescent layer, which seems resistant to hypochlorite treatment, is visibly thicker than in *D. majalis*. Our TEM images show that this layer remains mostly unaffected by the hypochlorite treatment. It could therefore be expected that this layer might be responsible for the interspecific difference in germination ability. We found that the permeability of carapace differs significantly between the tested species and that the proportion of permeable seeds is only slightly higher than the achieved germination percentage. This indicates that the carapace likely plays the role of a mechanical barrier preventing contact of water with the embryo and thereby preventing germination. More than 60 years ago, Veyret [36] hypothesized that the differences in in vitro germination ability between several European terrestrial orchid species might be caused by the carapace, because it is thicker in species which germinated poorly. Our results strongly support this assumption and indicate that carapace permeability may represent an important component of seed dormancy in orchids.

## Conclusions

The importance of chemical degradation of seed coats for improving germination has been observed empirically in numerous orchid species, although the mechanisms and especially the carapace have usually not been studied (e.g. [10, 17, 20, 24]). Besides chemical treatment, improvement of germination has also been achieved by mechanical scarification using vacuum [29], ultrasound [30, 31], mechanical removal of the testa [32], and by degradation of seed coats with enzymatic solutions: cellulase and maceroenzyme [29] or laccase [27]. Our data show that the impermeability of seed coats may represent an important component of seed dormancy in orchids and that the tiny carapace may be more important in this respect than the conspicuous testa. Seed dormancy caused by an impermeable seed coat is classified as Physical dormancy if it occurs alone, or as Combinational dormancy if it is combined with other mechanisms [37]. We cannot exclude the possibility of other factors, but our results clearly show for the first time that a physical or combinational dormancy may be involved in dust seeds. It is likely that the impermeability of the seed coats is present alongside endogenous mechanisms of dormancy in orchid seeds. The classification of orchid seeds into the classes Morphological dormancy or Morphophysiological dormancy, as proposed by Baskin et Baskin [37], therefore needs to be revisited, as at least some orchid species belong to Combinational dormancy. Further study should identify mechanisms that break this dormancy caused by the seed coat impermeability under natural conditions. The role of symbiotic fungi has been proposed, and it seems likely that they might be able to degrade seed coat layers with their enzymatic repertoire.

## Supplementary Information

Supplementary Material 1.

## Data Availability

The datasets used and/or analysed during the current study are available from the corresponding author on request.
