# Supplementary material for: Water permeability of seed coats determines differences in dormancy between two orchid species
Source: BMC Plant Biol. 2026 May 27;26:1306. doi: 10.1186/s12870-026-08982-0 (PMC13440099; doi:10.1186/s12870-026-08982-0)
Supplement: Supplementary file 1 [file 12870_2026_8982_MOESM1_ESM.docx]

| **species** | **origin** | **permission** | **germination response** |
| --- | --- | --- | --- |
| *Dactylorhiza majalis* (Rchb.) P.F.Hunt & Summerh. | plants naturalized in Prague Botanical Garden (originally collected in Rádlo, Liberec Region, Czech Republic, GPS 50.705N, 15.124E | SR/0065/KK/2023-6 | germinates relatively readily, even after very weak pretreatment |
| *Epipactis helleborine* (L.) Crantz | wild growing plants in spruce forests near Dářko, Vysočina Region, Czech Republic, GPS 49.620N, 15.900E | this species is not protected by the Czech Republic's laws, and collecting its seeds for scientific purposes does not require any permit under Czech law, see Act No. 114/1992 Sb. | germinates very poorly and requires intensive disinfection |

Supplementary table 1. Species used in the study.

Supplementary figure 1. Effect of different pretreatments with NaOCl solution on axenic *in vitro* seed germination of the *Epipactis helleborine*. Numbers on the x axis describe time of pretreatment with ethanol (EtOH) / time of pretreatment with CaOCl_2_ solution.

Supplementary figure 2. Effect of different pretreatments with H_2_SO_4_ solution on axenic *in vitro* seed germination of the *Epipactis helleborine*.
